# Processed Food Consumption Based on the NOVA Classification Is Associated With Elevated Cardiometabolic Risk in Turkish Adults

**DOI:** 10.1002/fsn3.71014

**Published:** 2025-09-26

**Authors:** Irem Ozkan, Tugce Ozlu Karahan, Hande Seven Avuk

**Affiliations:** ^1^ Department of Nutrition and Dietetics Institute of Graduate Programs, Istanbul Bilgi University Istanbul Türkiye; ^2^ Department of Nutrition and Dietetics Faculty of Health Sciences, Istanbul Bilgi University Istanbul Türkiye

**Keywords:** cardiometabolic risk, dietary habits, framingham risk score, obesity, processed food

## Abstract

The global rise in ultra‐processed food consumption is associated with increased prevalence of cardiometabolic disorders such as obesity, diabetes, and cardiovascular diseases. This study aimed to evaluate the relationship between processed food intake and cardiometabolic risk factors among adults in Türkiye. A cross‐sectional study was conducted among 171 adults aged 18–65 years attending an internal medicine outpatient clinic between March and April 2024. Data collected included dietary habits, physical activity levels, anthropometric measurements, and biochemical parameters. Processed food intake was classified according to the NOVA system and divided into tertiles. Cardiometabolic risk was assessed using the Framingham risk score. The median age was 44.0 (29.0–52.0) years; 67.9% were female. Participants in the T2 tertile with the highest processed food intake had a higher median waist/hip ratio [0.88; (0.82–0.90)] compared to those in the lowest tertile (T1) [0.82 (0.76–0.88); *p* = 0.003]. Similarly, the median Framingham risk score was higher in the highest tertile of T2 [11.0; (5.0–15.0)] compared to those in the lowest tertile [5.0; (−1.0–9.5); *p* < 0.001]. These differences suggest a positive correlation between processed food intake and cardiometabolic risk. Increased processed food consumption is associated with higher obesity and cardiometabolic risk among Turkish adults. Promoting healthy dietary habits and reducing processed food intake could have significant public health benefits. Future longitudinal studies with larger samples are needed to confirm these findings and clarify causality.

**Trial Registration:** ClinicalTrials.gov: NCT06996262

## Graphical abstract

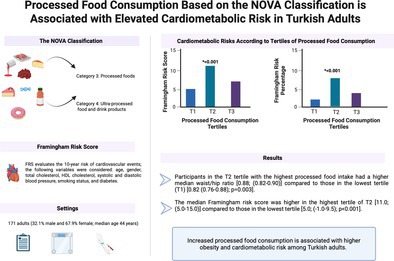

## 1 Introduction

Cardiovascular diseases (CVD) continue to be one of the leading causes of chronic disability and mortality worldwide. Cardiometabolic risk factors such as hypertension, dyslipidemia, obesity, and diabetes play a significant role in the development of these diseases. Poor nutrition is a modifiable key risk factor for CVD and represents a critical target in prevention efforts (Juul, Vaidean, Lin, et al. 2021; Mazur et al. 2024; Berisha et al. 2025). Current research, however, focuses more on overall dietary patterns and quality rather than a single nutrient in CVD prevention (Juul, Vaidean, and Parekh 2021).

The range of food processing—from minimally processed foods (e.g., frozen vegetables, dried fruits with no added sugar or additives, pasteurized milk) to ultra‐processed foods (UPFs) (e.g., carbonated soft drinks, fast food, industrially produced breads, sausage sandwiches)—can profoundly impact diet quality (Juul, Vaidean, and Parekh 2021). The production of UPFs involves a series of new processing techniques (e.g., extrusion), additives (e.g., modified starches, protein isolates), and industrially used special additives (e.g., emulsifiers, artificial flavors) (Monteiro et al. 2019). Processing can alter a food's health potential by removing beneficial nutrients and naturally occurring bioactive compounds, adding unhealthy nutrients and food additives, and changing physical structures (Juul, Vaidean, and Parekh 2021). UPF consumption has been associated with obesity, hypertension, metabolic syndrome, and type 2 diabetes (Juul et al. 2018; Elizabeth et al. 2020; Juul, Vaidean, and Parekh 2021; Rivera et al. 2024; Heidari Seyedmahalleh et al. 2024; Vallianou et al. 2025; Shim 2025). Although the evidence is still emerging, recent epidemiological studies suggest that higher UPF consumption is linked to increased CVD risk. In the 18‐year follow‐up of the Framingham Offspring Study, each additional daily UPF portion was associated with a 7% increase in CVD incidence risk (Juul, Vaidean, Lin, et al. 2021).

Worldwide, UPF consumption has increased exponentially. According to national dietary surveys, UPFs make up 25%–60% of total daily energy intake (Hosseininasab et al. 2022). According to the Türkiye Nutrition and Health Survey, the average daily energy intake in Türkiye was 1912.56 ± 736.36 kcal, of which 605.30 ± 422.69 kcal—corresponding to 30.64%—was derived from UPFs (Ministry of Health 2019; Aciduman Subasiay 2022). Türkiye is at a high risk regarding both UPF consumption and the prevalence of associated CVD. CVD has been the leading cause of all deaths in Türkiye, with rates of 40% in 1989, 45% in 1993, 40% in 2009, 38% in 2012, 39.5% in 2017, 37.8% in 2018, and 36.8% in 2019. Additionally, due to the aging population and rising rates of diabetes and obesity, it is estimated that CVD‐related mortalities will increase by approximately 2.3 times in men and 1.8 times in women by 2030. Türkiye currently has the highest rate of early myocardial infarction under age 50 in Europe (Tokgozoglu et al. 2021).

As production and consumption of UPFs have surged in recent years, understanding their potential health impacts has become an important concern for health systems. Given the high prevalence of CVD in Türkiye, identifying dietary factors that may be related to the disease is also an essential necessity. The primary aim of this study is to investigate the relationship between processed food intake and cardiometabolic risk factors among Turkish adults.

## 2 Materials and Methods

### 2.1 Study Design and Participants

Our cross‐sectional study was conducted among a sample of 171 volunteers aged 18–65 years who applied to the internal medicine outpatient clinic of a public hospital in Kocaeli, Türkiye, between March and April 2024. The sample size was calculated using G*Power 3.1.9.4 software, considering a one‐way ANOVA test based on similar studies. Sample size calculations indicated that with an effect size of 0.38 (based on Hosseininasab et al. 2022), a confidence level of 95% (alpha = 0.05), and a power of 95%, at least 108 individuals were required. Exclusion criteria included individuals under 18 years of age, over 65, pregnant and breastfeeding women, and individuals with active cancer or chronic illnesses. The study's ethical approval was obtained from the Human Research Ethics Committee of Istanbul Bilgi University, prepared in accordance with the Helsinki Declaration's ethical standards (Approval No: 2024‐20160‐015, Date: 29.01.2024). Written consent was obtained from all participants after verbal information. This research is registered with ClinicalTrials.gov under the number NCT06996262.

### 2.2 Data Collection

**FIGURE 1:**
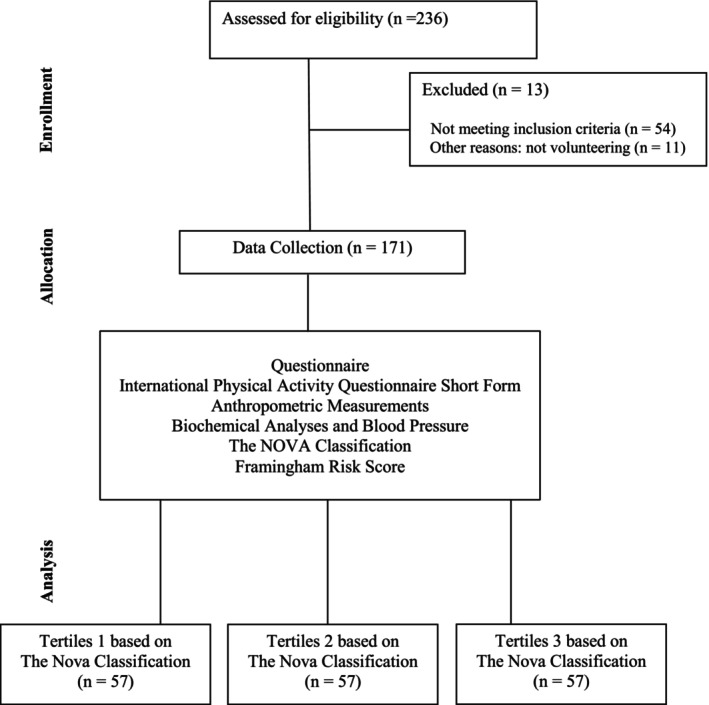
CONSORT flow diagram of the study.

The questionnaire consisted of structured questions covering sociodemographic information, health status, dietary habits, and physical activity levels. The survey was conducted through face‐to‐face interviews by the researcher, and all responses were recorded directly by the researcher. Following the questionnaire, anthropometric measurements were taken, and blood samples were collected for biochemical analyses. The Consort Flow Diagram of the study is shown in Figure 1.

#### 2.2.1 Physical Activity Level

Physical activity level was assessed using the International Physical Activity Questionnaire Short Form (IPAQ). This questionnaire queries individuals aged 15–65 years about their walking, moderate, vigorous activities, and sedentary time over the past week (Craig et al. 2003) and has been validated among Turkish (Savcı et al. 2006). The questionnaire consists of a total of seven questions. The first six questions inquire about physical activities performed for at least 10 min within the last 7 days. The final question assesses the amount of time spent sitting or lying down. The metabolic equivalent (MET) was used in evaluating IPAQ. The MET total was calculated by multiplying the type of physical activity performed, the number of days it was performed, and the duration of each activity (IPAQ Research Committee 2004).

#### 2.2.2 Anthropometric Measurements

Participants' body measurements were obtained, including body weight, height, waist circumference, and hip circumference. Height was measured using a stadiometer, with participants standing barefoot, their feet together, and their heads positioned in the Frankfurt horizontal plane. Body weight was measured with a calibrated portable digital scale (Tanita bc 601), which was accurate to 50 g. Waist and hip circumferences were measured with a non‐stretchable measuring tape. The body mass index (BMI) was calculated by dividing the body weight in kilograms by the square of height in meters (kg/m^2^). According to the World Health Organization classification, BMI was categorized as follows: underweight (< 18.5 kg/m^2^), normal weight (18.5–24.9 kg/m^2^), overweight (25.0–29.9 kg/m^2^), and obese (≥ 30 kg/m^2^) (WHO 2010).

#### 2.2.3 Biochemical Analyses and Blood Pressure

Biochemical parameters of the participants were assessed using blood samples collected after 12 h of fasting. Lipid profiles—including total cholesterol, HDL, LDL, and triglycerides—and glucose, HbA1c, and uric acid levels were analyzed using routine methods in the hospital laboratory. Blood pressure was measured by a nurse using a standard cuff sphygmomanometer.

#### 2.2.4 The NOVA Classification

Daily processed food intake was calculated according to the NOVA classification, based on food consumption frequency and considering standard portion sizes. The University of São Paulo in Brazil developed the NOVA classification and is recognized by the Food and Agriculture Organization (FAO) of the United Nations as a valid tool for public health research and policy (Sadler et al. 2021). This classification system categorizes foods based on the nature, extent, and purpose of their industrial processing, thereby reflecting overall food quality. According to this system, foods are divided into four groups:

- Group 1: Unprocessed or minimally processed foods
- Group 2: Processed culinary ingredients
- Group 3: Processed foods
- Group 4: Ultra‐processed foods

According to the NOVA classification, daily processed food consumption was calculated using participants' self‐reported consumption frequencies for processed (Group 3) and ultra‐processed (Group 4) food items. Participants were asked to indicate their food consumption frequency as never, once a month, once every 2 weeks, 1–2 times a week, 3–4 times a week, 5–6 times a week, or every day. National standard portion sizes determined in the Turkey Nutrition Guide (TÜBER) 2022 (Ministry of Health 2022) were used as a reference for estimating consumption frequency. For example, a glass of milk is 200 mL, a slice of white bread is 25 g, and a bowl of ready‐made soup is 200 mL. For a better understanding of portion concepts, visual examples from food atlases were provided for each food group. This approach ensured that participants' responses were recorded according to standard portion sizes based on national guidelines. The researcher recorded all responses to the questionnaire, and daily intake in each NOVA group was calculated based on these responses. Interval frequency expressions such as ‘3–4 times a week’ were preferred because they more realistically reflect participants' consumption habits. To avoid ambiguity when including such intervals in the analysis, the arithmetic mean of each category was calculated and converted to daily serving equivalents. For example, ‘3–4 times a week’ was calculated as [(3 + 4)/2] = 3.5/7 = 0.5 servings/day. Based on the NOVA scores, participants were divided into tertiles according to the number of processed food servings they consumed daily: first tertile (≤ 5.07 servings), second tertile (5.08–7.61 servings), and third tertile (≥ 7.62 servings).

#### 2.2.5 Framingham Risk Score

The Framingham Risk Score (FRS), created by D'Agostino et al. (2008), was used to assess CVD risk in participants. To assess 10‐year cardiovascular risk, each participant is assigned a total risk score based on factors such as age, sex, total cholesterol level, HDL cholesterol level, systolic blood pressure (depending on whether the participant is receiving antihypertensive treatment), smoking status, and diabetes status. For example, for a 55‐year‐old man with a total cholesterol level of 240 mg/dL, HDL cholesterol of 45 mg/dL, systolic blood pressure of 140 mmHg (under antihypertensive treatment), who does not smoke and does not have diabetes:

- Age (55–59 years): 10 points
- Total cholesterol (240 mg/dL): 3 points
- HDL cholesterol (45 mg/dL): 0 points.
- Systolic blood pressure (140 mmHg, treated): 4 points
- Smoking: None = 0 points
- Diabetes: None = 0 points
- Total score: 17 points

This score corresponds to an estimated 10‐year cardiovascular risk of 29.4%. A higher score indicates a higher cardiometabolic risk.

### 2.3 Statistical Analysis

The data for this study were analyzed using the Statistical Package for the Social Sciences (SPSS) version 25.0 for Windows. Descriptive statistics included counts, percentages, medians, and the 25th and 75th percentiles. The Kolmogorov–Smirnov test indicated that continuous variables did not follow a normal distribution. The comparison of parameters across tertiles of processed food consumption was performed using the Kruskal–Wallis test. The relationship between categorical variables was examined with the chi‐square test. To facilitate advanced analyses and to reduce the impact of outliers, variables were transformed using a logarithmic transformation to achieve a more normal distribution suitable for parametric testing. To assess the effects of processed food consumption on the Framingham risk percentage (FRP), multivariable linear regression models were developed, incorporating variables such as gender, BMI, physical activity, income status, education level, and employment status. A multivariable linear regression model was also constructed to identify independent factors affecting processed food consumption. Statistical significance was set at *p* < 0.05.

## 3 Results

The sample of our study consisted of 171 adults with a median age of 44.0 years (29.0–52.0); 32.1% were male and 67.9% female. When examining the general characteristics of the participants, the median body weight, BMI, and waist circumference were found to be 75.0 kg (65.0–85.0), 26.3 kg/m^2^ (23.4–30.4 kg/m^2^), and 95.0 cm (85.0–102.0 cm), respectively. Participants in the first tertile had a median waist‐to‐hip ratio of 0.82 (0.76–0.88), while those in the second tertile had a ratio of 0.88 (0.82–0.90), with this difference being statistically significant (*p* = 0.003). Regarding gender, 30.2% of women and 40.0% of men were in the highest tertile of processed food consumption (*p* = 0.039). There were no significant differences among groups based on education level, employment status, income level, presence of chronic disease, or physical activity level (*p* > 0.05) (Table 1).

**TABLE 1:** General characteristics of participants by tertiles of processed food consumption.

	T1 (≤ 5.07; *N* = 57)	T2 (5.08–7.61; *N* = 57)	T3 (≥ 7.62; *N* = 57)	Total (*n* = 171)	*p*
Age (year)	36.0 (25.0–50.5)	48.0 (41.0–56.5)	41.0 (30.5–50.5)	44.0 (29.0–52.0)	**< 0.001**
Body weight (kg)	78.0 (62.5–90.0)	79.0 (70.5–89.5)	70.0 (65.0–80.0)	75.0 (65.0–85.0)	0.058
BMI (kg/m^2^)	26.4 (23.1–30.9)	26.8 (24.4–30.8)	26.2 (23.2–27.9)	26.3 (23.4–30.4)	0.496
Waist circumference (cm)	90.0 (75.0–100.0)	96.0 (86.5–104.0)	93.0 (84.5–103.0)	95.0 (85.0–102.0)	0.094
Waist/Hip ratio	0.82 (0.76–0.88)	0.88 (0.82–0.90)	0.84 (0.82–0.89)	0.85 (0.79–0.89)	**0.003**
Neck circumference (cm)	34.0 (32.2–36.0)	35.0 (33.5–37.0)	35.0 (33.0–36.0)	34.0 (33.0–36.0)	0.457
Waist/Height ratio	0.52 (0.44–0.59)	0.55 (0.50–0.64)	0.55 (0.51–0.60)	0.55 (0.48–0.60)	0.114
Gender					
Female	46 (39.7)	35 (30.2)	35 (30.2)	116 (67.8)	**0.039**
Male	11 (20.0)	22 (40.0)	22 (40.0)	55 (32.2)	
Educational status					
High school and below	36 (31.6)	39 (34.2)	39 (34.2)	114 (66.7)	0.789
Bachelor's degree and above	21 (36.8)	18 (31.6)	18 (31.6)	57 (33.3)	
Working status					
Yes	23 (29.9)	32 (41.6)	22 (28.6)	77 (45.0)	0.116
No	34 (36.2)	25 (26.6)	35 (37.2)	94 (55.0)	
Income status					
≤ Minimum wage	18 (26.9)	23 (34.3)	26 (38.8)	67 (39.2)	0.301
> Minimum wage	39 (37.5)	34 (32.7)	31 (29.8)	104 (60.8)	
Chronic disease status					
Yes	27 (39.1)	23 (33.3)	19 (27.5)	69 (40.4)	0.312
No	30 (29.4)	34 (33.3)	38 (37.3)	102 (59.6)	
Physical activity level					
Low	6 (35.3)	4 (23.5)	7 (41.2)	17 (9.9)	0.745
Moderate	38 (31.1)	43 (35.2)	41 (33.6)	122 (71.3)	
High	13 (40.6)	10 (31.3)	9 (28.1)	32 (18.7)	

*Note:* Continuous data are shown as median (25–75th percentile), and categorical variables are shown as number (percentage). One‐way ANOVA was applied for continuous data, and the chi‐square test was applied for categorical variables. Statistical significance (*p* < 0.05) is indicated in bold.

Abbreviation: BMI, body mass index.

Cardiometabolic risk according to participants' tertiles of processed food consumption was examined in Table 2. Systolic and diastolic blood pressures showed significant differences between tertiles (*p* = 0.021 and *p* = 0.001, respectively). Participants in the first tertile had significantly lower diastolic blood pressure values [74.0 mmHg (68.0–78.5)] compared to those in the second [79.0 mmHg (71.0–90.0)] and third tertiles [79.0 mmHg (70.5–90.5)]. However, differences in systolic blood pressure were only observed between the first and second tertiles. The median FRS for participants in the first tertile was 5.0 (−1.0 to 9.5), while in the second tertile it was 11.0 (5.0–15.0), and this difference was statistically significant (*p* < 0.001). Similar differences were found for the FRP (*p* < 0.001). Although LDL cholesterol levels increased across the first three tertiles of processed food consumption, the differences were not statistically significant (*p* = 0.992). No other parameters showed significant differences between groups (*p* > 0.05) (Table 2).

**TABLE 2:** Evaluation of participants' cardiometabolic risks according to tertiles of processed food consumption.

	T1 (≤ 5.07; *N* = 57)	T2 (5.08–7.61; *N* = 57)	T3 (≥ 7.62; *N* = 57)	Toplam (*n* = 171)	*p*
SBP (mm/Hg)	108.0 (102.0–113.5)	114.0 (103.0–126.0)	112.0 (102.0–123.0)	110.0 (102.0–120.0)	**0.021**
DBP (mm/Hg)	74.0 (68.0–78.5)	79.0 (71.0–90.0)	79.0 (70.5–90.5)	76.0 (70.0–86.0)	**0.001**
Glucose (mg/dL)	96.0 (88.0–105.0)	100.0 (90.3–115.6)	97.0 (90.09–106.5)	97.6 (89.6–109.0)	0.378
Total cholesterol (mg/dL)	197.0 (167.2–221.5)	194.8 (164.9–222.5)	192.5 (158.8–209.9)	193.0 (163.5–220.0)	0.561
HDL cholesterol (mg/dL)	54.2 (45.0–62.4)	49.7 (39.6–57.5)	52.0 (42.8–61.4)	52.0 (43.0–59.1)	0.086
LDL cholesterol (mg/dL)	115.0 (88.2–136.3)	117.0 (95.0–138.4)	121.3 (87.5–137.2)	117.0 (90.8–138.4)	0.992
Triglyceride (mg/dL)	120.0 (89.4–159.4)	132.0 (93.5–179.0)	110.0 (77.5–162.8)	119.7 (88.2–164.0)	0.428
HbA1C (%)	5.3 (5.1–5.8)	5.6 (5.1–6.1)	5.6 (5.1–5.8)	5.4 (5.1–5.9)	0.103
Uric acid (mg/dL)	3.9 (3.3–4.7)	4.2 (3.2–5.5)	3.6 (3.2–4.9)	3.9 (3.2–5.0)	0.481
FRS	5.0 (−1.0–9.5)	11.0 (5.0–15.0)	8.0 (1.5–15.0)	8.0 (2.0–13.0)	**< 0.001**
FRP	2.0 (1.0–5.5)	8.0 (2.0–20.0)	4.0 (1.0–20.0)	4.0 (1.0–12.0)	**< 0.001**

*Note:* Continuous data are presented as median (25th–75th percentile). Statistical significance was determined by one‐way ANOVA (*p* < 0.05), and significant values are indicated in bold.

Abbreviations: DBP, diastolic blood pressure; FRP, Framingham risk percentage; FRS, Framingham risk score; SBP, systolic blood pressure.

The multivariable linear regression models applied to examine the effect of processed food consumption on FRP are presented in Table 3. In the first model (adjusted for gender), it was determined that processed food consumption had no significant effect on FRP (*p* = 0.033). When BMI was added to the initial model, followed by the inclusion of physical activity level, a positive and significant effect of processed food consumption on FRP was observed (*p* = 0.042 and *p* = 0.046, respectively). Each unit increase in processed food consumption was associated with an average increase of 0.374% in FRP in Model 2 (*p* = 0.042) and 0.368% in Model 3 (*p* = 0.046). Finally, after adding education level, employment status, and income level to the model, the significance of processed food consumption disappeared (*p* = 0.063) (Table 3).

**TABLE 3:** Multivariable linear regression models examining the effect of processed food consumption on Framingham risk percentage.

	Framingham risk percentage				
	β	*T*	95% confidence interval		*p*
			Lower	Upper	
Processed food consumption					
Crude	0.403	2.153	0.033	0.773	**0.033**
Model 1	0.354	1.892	−0.015	0.724	0.060
Model 2	0.374	2.053	0.014	0.733	**0.042**
Model 3	0.368	2.014	0.007	0.729	**0.046**
Model 4	0.344	1.868	−0.021	0.708	0.063

*Note:* Model 1: Adjusted for gender (*R* ^2^: 0.051; 0.012). Model 2: Adjusted for gender, BMI (*R* ^2^: 0.109; *p* < 0.001). Model 3: Adjusted for gender, BMI, physical activity (*R* ^2^: 0.111; *p* < 0.001). Model 4: Adjusted for gender, BMI, physical activity, employment status, education level, income status (*R* ^2^: 0.148; *p* < 0.001). Statistical significance (*p* < 0.05) is indicated in bold.

The multivariable linear regression model assessing the effect of independent variables on processed food consumption is shown in Table 4. Among the independent variables included in the model, age (β = 0.326; *p* = 0.009), gender (β = −0.100; *p* = 0.015), and income level (β = −0.078; *p* = 0.023) were identified as determinants of processed food consumption (*R* ^2^ = 0.113; *p* = 0.011, Figure 1).

**TABLE 4:** Multivariable linear regression model of determinants of processed food consumption.

	Processed food consumption				
	β	*T*	95% confidence interval		*p*
			Lower	Upper	
Age (continuous)	0.326	2.647	0.083	0.568	**0.009**
BMI (continuous)	−0.356	−1.537	−0.813	0.101	0.126
Gender					
Male (reference)	—	—	—	—	—
Female	−0.100	−2.459	−0.180	−0.020	**0.015**
Physical activity level					
Low (reference)	—	—	—	—	—
Modarete and high (active)	−0.041	−0.724	−0.152	0.070	0.470
Income status					
≤ Minimum wage (reference)	—	—	—	—	—
> Minimum wage	−0.078	−2.299	−0.145	−0.011	**0.023**
Eğitim durumu					
High school and below (ref reference)	—	—	—	—	—
Bachelor's degree and above	−0.051	−1.224	−0.133	0.031	0.223
Chronic disease status					
Yes (reference)	—	—	—	—	—
No	0.061	1.796	−0.006	0.128	0.074

*Note:* *R* ^2^: 0.113; *p* = 0.011. Statistical significance (*p* < 0.05) is indicated in bold.

## 4 Discusiıon

In recent years, notable changes in dietary habits have occurred, particularly a significant increase in processed and ultra‐processed foods (UPF) consumption. This trend poses a serious threat to public health, associated with a parallel rise in the prevalence of cardiometabolic diseases (Donat‐Vargas et al. 2021; Juul, Vaidean, and Parekh 2021). In this context, evaluating the impact of processed food consumption on cardiometabolic risk among Turkish adults is of great importance for developing effective public health policies.

In our study, it was found that an increase in processed food consumption was significantly associated with higher FRS and FRP. Our findings are consistent with previous studies indicating that healthier dietary models containing less processed food are linked to lower cardiovascular risk (Shan et al. 2020; Juul, Vaidean, Lin, et al. 2021; Satija et al. 2017). The NutriNet Santé cohort study, conducted among 105,159 individuals, identified that each 10% increase in the proportion of UPFs in the diet was associated with increases of approximately 12%, 13%, and 11% in overall cardiovascular disease, coronary heart disease, and cerebrovascular disease rates, respectively (Srour et al. 2019). The biological pathways through which UPFs affect cardiovascular health may include interactions among many components and properties of UPFs that are not yet fully understood. Key mechanisms involve changes in serum lipid concentrations, alterations in gut microbiota composition and host–microbe interactions, and links to obesity, inflammation, oxidative stress, insulin resistance, hypertension, and hormonal imbalances (Juul, Vaidean, and Parekh 2021). Increased processed food consumption promotes visceral fat accumulation, thereby triggering obesity, an important cardiometabolic risk. A 10% increase in processed food intake has been associated with higher obesity incidence (Beslay et al. 2020). Epidemiological and experimental studies have linked higher UPF consumption with excessive fat accumulation (Juul et al. 2018; Poti et al. 2017). Two cohort studies from Spain and Brazil found that adults in the highest quartile of UPF intake had approximately 26%–29% higher risk of being overweight over follow‐up periods of about 9 and 4 years, respectively, compared to those in the lowest quartile (de Deus Mendonca et al. 2016; Canhada et al. 2020). UPFs may contribute to weight gain through dietary patterns characterized by high fat, salt, sugar, and artificial sweeteners, which make UPFs highly palatable and can replace endogenous satiety mechanisms (Juul, Vaidean, and Parekh 2021). Although no significant relationship was observed between daily processed food intake and BMI in our study, the waist‐hip ratio increased significantly with higher processed food consumption. The differences in FRS, waist‐hip ratio, and blood pressures between the first and second tertiles may be related to the fact that the median age, an independent risk factor, was higher among participants in the second tertile.

Since processed foods are rich in trans fats and saturated fats, their consumption negatively impacts the lipid profile, leading to an increased prevalence of dyslipidemia, a cardiometabolic risk factor (Donat‐Vargas et al. 2021). Additionally, the increase in daily processed food intake is thought to impair blood sugar regulation, thereby elevating cardiometabolic risk factors. Literature indicates that processed food consumption raises the risk of dyslipidemia, which is a known cardiometabolic risk factor. In a study conducted by Donat‐Vargas et al. (2021) with 1821 participants, individuals with high processed food consumption had elevated triglyceride levels and lower HDL cholesterol levels (Donat‐Vargas et al. 2021). Similarly, Scaranni and colleagues observed in their study in Brazil that high processed food intake increased the prevalence of dyslipidemia. However, some studies have not found a relationship between LDL cholesterol and processed food consumption (Scaranni et al. 2021). The Brazil Dietary and Nutrition Survey conducted between 2008 and 2009 found that processed food intake increased blood glucose levels (Magalhães et al. 2022). Moreover, Duan et al. (2022) reported that a 10% increase in processed food consumption was associated with a 25% higher risk of developing type 2 diabetes.

In our study, unlike the literature, no statistically significant differences were found between fasting blood glucose, HDL cholesterol, LDL cholesterol, triglycerides, total cholesterol, and processed food consumption. Since the relationship between biochemical parameters and processed food intake may develop over time, this relationship might not be detectable with single‐time measurements.

Hypertension is also an important risk factor for cardiovascular disease (CVD) and stroke. Epidemiological studies support that higher sodium‐potassium ratios (≥ 1.0) are associated with increased CVD mortality risk, whereas higher potassium intake is associated with reduced risk (O'Donnell et al. 2014). Because commercially processed foods are the primary source of sodium in the diet, increased UPF consumption can alter the sodium‐potassium ratio, potentially affecting CVD risk (Juul, Vaidean, and Parekh 2021). In our study, consistent with these findings, systolic and diastolic blood pressures increased as we moved from the first to the second tertile of processed food consumption.

In our study, processed food consumption was examined regarding sociodemographic characteristics, and it was found that male gender was associated with higher processed food intake. Similarly, Rauber et al. (2020) conducted a study in 2020 in England with 6143 participants aged 19–90, and they observed that men consumed significantly more processed foods than women. The higher proportion of men in our sample, who are likely to spend more time outside of work, may contribute to increased processed food consumption. Individuals with higher education levels tend to be more conscious about nutrition and prefer healthier foods over processed options (Sprake et al. 2018). Between 2017 and 2019, Chen and colleagues investigated the relationship between processed food consumption and European education level. Their study, involving 20,328 participants, found that individuals with lower education levels consumed more processed foods (Chen et al. 2020). Developing targeted interventions and health policies for populations with lower education levels aims to improve public health (Marchese et al. 2021). In our study, most participants in the third tertile had a high school education or below, but these differences were not statistically significant. There is a complex relationship between income level and processed food consumption. While individuals with lower income may prefer processed foods to save costs and durability, those with higher income have access to a broader range of products and may consume more processed foods due to busy lifestyles (Marino et al. 2021). French and colleagues identified higher processed food consumption levels in high‐income countries. Wealthier countries have broader product ranges and faster‐paced lifestyles, promoting processed food intake (French et al. 2019). Interestingly, in our study, lower income levels were associated with higher processed food consumption. In our study, it was determined that low income level increases the tendency toward processed food consumption.

This study possesses several notable strengths. The combined evaluation of self‐reported dietary surveys alongside biochemical parameters and anthropometric measurements in our research enhances the reliability of the findings. Furthermore, using the NOVA classification when assessing processed food consumption and calculating cardiometabolic risk using a reliable tool such as the FRS bolsters the study's methodological rigor. Nevertheless, despite the study's significant findings, some limitations should be considered. The cross‐sectional design of the research and the self‐reported assessment of processed food consumption are important limitations. Additionally, the restriction of the sample to individuals admitted to a single hospital limits the generalizability of the findings.

## 5 Conclusion

This study evaluated the association between processed food consumption and cardiometabolic risk factors among adult individuals living in Türkiye. The findings indicate a significant increase in the waist‐hip ratio and the FRP and FRS with higher daily processed food consumption. Notably, the high consumption of ultra‐processed foods emerges as a dietary habit that can negatively impact cardiometabolic health, particularly obesity. However, larger and longer‐term studies are needed to elucidate processed foods' effects fully. Furthermore, our study found that older age, male gender, and low‐income level impact processed food consumption. Accordingly, educational and policy interventions planned by considering these sociodemographic factors to reduce the community's processed food consumption could be an important strategy in preventing cardiovascular diseases.

## Author Contributions

**Irem Ozkan:** conceptualization (equal), data curation (equal), formal analysis (equal), investigation (equal), methodology (equal), resources (equal), software (equal), writing – original draft (equal). **Tugce Ozlu Karahan:** formal analysis (equal), software (equal), visualization (equal), writing – original draft (equal), writing – review and editing (equal). **Hande Seven Avuk:** conceptualization (equal), formal analysis (equal), investigation (equal), methodology (equal), project administration (equal), supervision (equal), visualization (equal), writing – original draft (equal), writing – review and editing (equal).

## Disclosure

The authors have nothing to report.

## Ethics Statement

Ethical approval for this study was obtained from the Human Research Ethics Committee of Istanbul Bilgi University, prepared in accordance with the Helsinki Declaration's ethical standards (Approval No: 2024‐20160‐015, Date: 29.01.2024). Informed consent was obtained from all individual participants included in the study.

## Conflicts of Interest

The authors declare no conflicts of interest.

## Data Availability

The data that support the findings of this study are available from the corresponding author upon reasonable request.
